# Cornel Iridoid Glycoside Improves Locomotor Impairment and Decreases Spinal Cord Damage in Rats

**DOI:** 10.1155/2016/6725381

**Published:** 2016-11-20

**Authors:** Wen-jing Tang, Deng-lei Ma, Cui-cui Yang, Li Zhang, Ya-li Li, Lan Zhang, Lin Li

**Affiliations:** Department of Pharmacology, Xuanwu Hospital of Capital Medical University, Beijing Institute for Brain Disorders, Beijing Engineering Research Center for Nerve System Drugs, Key Laboratory for Neurodegenerative Diseases of Ministry of Education, Beijing 100053, China

## Abstract

*Purpose.* This study was to investigate the effects of cornel iridoid glycoside (CIG) on spinal cord injury (SCI) in rats.* Methods.* The thoracic cord (at T9) of rats was injured by clip compression for 30 sec. Locomotor function was assessed using the Basso, Beattie, and Bresnahan (BBB) rating scale. Neuroanatomic stereological parameters as well as Nogo-A, p75 neurotrophin receptor (p75NTR), and ROCKII expression were measured by histological processing, immunohistochemistry, and stereological analyses. The axons passing through the lesion site were detected by BDA tracing.* Results.* Intragastric administration of CIG (60 and 180 mg/kg) improved the locomotor impairment at 10, 17, 24, and 31 days post-injury (dpi) compared with untreated SCI model rats. CIG treatment decreased the volume of the lesion epicenter (LEp) and increased the volume of spared tissue and the number of surviving neurons in the injured spinal cord at 31 dpi. CIG promoted the growth of BDA-positive axons and their passage through the lesion site and decreased the expression of Nogo-A, p75NTR, and ROCKII both in and around the LEp.* Conclusion.* CIG improved the locomotor impairment, decreased tissue damage, and downregulated the myelin-associated inhibition signaling pathway in SCI rats. The results suggest that CIG may be beneficial for SCI therapy.

## 1. Introduction

Spinal cord injury (SCI) is a severe, often life-threatening, and debilitating clinical condition, affecting mainly young people, and the treatment incurs massive costs associated with ongoing care. This makes SCI a serious social and economic problem [1, 2]. The pathophysiological events that follow an insult to the spinal cord are customarily categorized into primary and secondary stages, followed by a chronic phase [3, 4]. Besides the immediate cell death in the primary stage, the second stage consists of a cascade of vascular, inflammatory, biochemical, and glial processes, causing neuron death and progressive degeneration [5, 6].

SCI pathogenesis also involves the myelin-associated inhibition signaling pathway that plays an important role in restricting axonal growth and neurological recovery [7]. Nogo-A inhibits axon/myelin regeneration and is mainly distributed in oligodendrocyte cell bodies and their myelin sheaths in the nerve fiber tracts of the central nervous system (CNS) [8, 9]. Nogo-A transduces signals to the neurons through the p75 neurotrophin receptor (p75NTR) and Nogo receptor (NgR) [10] and then activates Rho-associated coiled-coil forming protein kinase (ROCK) (mainly ROCKII) [11, 12], leading to growth cone collapse and inhibition of neurite/axon outgrowth [13, 14].

*Cornus officinalis* is a member of the Cornaceae family and was first recorded in Shennong's Classic of Materia Medica about 2000 years ago in China. In traditional Chinese medicine,* Cornus officinalis* is used to tonify the liver and the kidney for the treatment of vertigo, aching back, spontaneous emission, and sweating [15].* Cornus officinalis* is also used to treat stroke in combination with other herbs [16]. Cornel iridoid glycoside (CIG) is the major component extracted from the sarcocarp of* Cornus officinalis*. In the previous studies, we found that CIG inhibited inflammation and apoptosis, promoted neurogenesis and angiogenesis in the brain, and improved neurological deficits after focal cerebral ischemia in rats [17, 18]. We also discovered that CIG treatment enhanced neurotrophic factor expression, promoted neuronal survival in the brain, and ameliorated memory impairment in a brain injury rat model induced by fimbria-fornix transection [19]. However, whether CIG has a beneficial effect on SCI remains unknown.

In the present study, we used a clip compression method to establish the SCI rat model, which is considered to be a suitable model for SCI studies and related therapeutic interventions [20, 21]. We investigated the effects of CIG on the locomotor function and the stereological parameters of the spinal cord neuroanatomical structure 31 days after SCI surgery. To further explore the underlying mechanisms, we observed the expression of important molecules, including Nogo-A, p75NTR, and ROCKII, in the myelin-associated inhibition signaling pathway in the injured spinal cord.

## 2. Methods

### 2.1. Drugs

Cornel iridoid glycoside (CIG) was extracted from the sarcocarp of* Cornus officinalis*, which was purchased from Tong Ren Tang Company, Beijing, China. The technique of extraction was developed by our laboratory and described briefly in a previous paper by Yao et al. [18]. The purity of CIG was 71.19% as tested by RP-HPLC. Morroniside constituted 67% of the CIG, while loganin constituted 33% in CIG.

### 2.2. Animals and Experimental Grouping

Adult female Wistar rats (7-8 weeks of age, weight of 200–220 g) were obtained from Beijing Vitalriver Experimental Animal Co. (Beijing, China). They were housed in a controlled environment with a temperature at 24 ± 1°C, a 12/12 h dark/light cycle, with the humidity maintained at 60% to 70%. During the whole experiment, rats were free to get food and water. All experiments were done according to the rules of the Provisions and General Recommendations of Chinese Experimental Animal Administration Legislation.

Rats were randomly divided into 4 groups (*n* = 12 in each group): the sham control group, the SCI model group, SCI+CIG 60 mg/kg group, and SCI+CIG 180 mg/kg group. CIG was dissolved in normal saline and administered intragastrically starting at 4 h after the surgery of SCI and once a day for 31 days. The sham control and the model groups received an equal volume of normal saline.

### 2.3. Spinal Cord Injury Surgery

**Figure 1:**
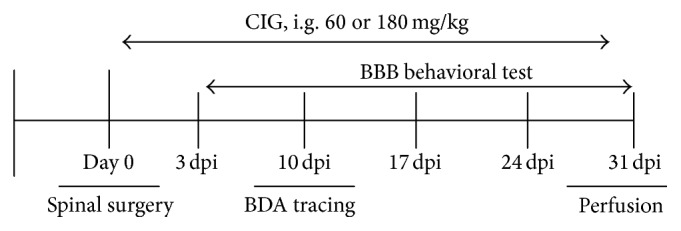
Flow chart of experimental schedule.

Figure 1 shows the timeline of experimental design in general. After being fasted for 12 h, rats were anesthetized with 10% chloral hydrate (0.4 ml/kg, i.p.). A laminectomy was made to expose thoracic vertebrae 9 (T9) after a midline incision on the skin and separation of the muscles. A vessel clip (Müller temporary, FE011K, 50 g closing force, Aesculap AG & Co. KG, Tuttlingen, Germany) was applied to the spinal segment T9 for 30 sec to induce the compression injury [22]. The sham control group was subjected to only a laminectomy procedure without compression to the spinal cord. A small piece of sterile surgical gel-foam was fixed over the laminectomy after irrigating with saline solution. The musculature and the skin were sewed up with absorbable sutures. Body temperature was maintained with a heating pad at 37°C during the surgery and postoperative period. The bladders of rats were expressed manually twice a day until bladder function returned.

### 2.4. BDA Tracing Surgery

At 10 days post-injury (dpi), 3 rats in each group were selected randomly for biotinylated dextran amine (BDA) tracing surgery. The animals were anesthetized and positioned on a stereotaxic apparatus. A dental drill was used to make burr holes through the cranium bilaterally overlying the sensorimotor cortex (coordinates: 2 mm posterior to Bregma, 2 mm lateral to Bregma, and 1.5 mm depth) [23] and at the red nucleus (coordinates: 5.6 mm posterior to Bregma, 0.9 mm lateral to Bregma, and 7.4 mm depth) [24]. Five microliters of 10% BDA (Molecular Probes, Invitrogen, USA) was injected sequentially through each of the holes described above at a 0.2 *μ*l/min flow rate, using a 5 *μ*l, 24 g Hamilton syringe model 7105 (Hamilton Company, NV, USA) set on a syringe pump (Kd Scientific Holliston, MA, USA). The syringe remained for an additional 5 min before retraction as described before [25]. A thin layer of aseptic bone wax was used to cover the holes prior to a subdermal suture closure of the skin. Except for the BDA injections, the treatment and dosages for administration remained the same as before till the end of the observation period at 31 dpi.

### 2.5. Behavioral Assessment

The behavioral assessment was performed weekly at 3, 10, 17, 24, and 31 dpi, respectively. Before the surgery, the animals were trained over a period of 5 days for acquaintance with the environment and technicians as preparation for the behavioral test. The open-field locomotor test was conducted by using the 21-point Basso, Beattie, and Bresnahan (BBB) locomotor rating scale [26]. The rats were placed in an open field (120 cm in diameter, 80 cm in height) with a nonslippery floor. In all testing sessions, the animals were observed individually for at least 4 min by two investigators blind to the animal grouping.

### 2.6. Perfusion and Tissue Preparation

For the histological analysis of the spinal cord, all rats were anesthetized with 10% chloral hydrate (0.4 ml/kg, i.p.) and perfused transcardially with 0.01 M PBS (pH 7.4). The spinal cords were removed and postfixed in 4% paraformaldehyde in 0.01 M PBS for 72 h and then switched to 30% sucrose/0.1 M PBS. The segment was selected for analysis from rostral to caudal areas in which the lesion epicenter was on center and throughout the entire injury (defined by anatomical T7–T11 spinal roots, approximately 15 mm long). All the segments were moved and embedded in optimum cutting temperature compound at −20°C. After being frozen in isopentane, each segment was cut into serial, 30 *μ*m thick, and horizontal sections in a cryostat for the stereological histological analyses, and then two sections from every eighth section were directly mounted on gelatin-coated slices for myelin basic protein (MBP) and immunohistochemistry staining.

### 2.7. Histology and Immunohistochemistry

In order to measure the stereological parameters of spinal cord lesion, we used MBP for presentation of myelin and counterstained with cresyl violet for Nissl substance [27, 28]. Before processing, sections were dried for a minimum of 2-3 h at room temperature. Afterward, they were washed in 0.01 M PBS (pH 7.4) and then incubated in 3% hydrogen peroxide/methanol for 15 min. After rinsing three times by PBS, sections were exposed overnight to MBP (1 : 1000 dilution, Merck Millipore, Germany). The next day, sections were washed in PBS and then incubated in ChemMate™EnVision+/horseradish peroxidase (HRP, Number A from EnVision™ Detection Kit, Gene Tech, Shanghai, China) for 30 min in a humidified chamber. After several PBS rinses, the signal was visualized with diaminobenzidine (DAB) substrate buffer (Number B from EnVision Detection Kit). The sections were then placed in cresyl violet for 30 min after the DAB reaction was terminated in distilled water. After washing in distilled water, the sections were differentiated in 95% ethanol for 15 sec and then in 100% alcohol twice for 30 sec each. Subsequently, the sections were defatted in xylene three times for 2 min each and coverslipped with neutral balsam.

For the detection of Nogo-A, p75NTR, and ROCKII, randomly chosen slide series mounted with evenly spaced tissue sections were processed for immunohistochemical staining. The sections were incubated in 3% H_2_O_2_/methanol for 15 min and then exposed overnight to the primary antibodies including anti-Nogo-A (1 : 500, Santa Cruz Biotechnology, TX, USA), anti-p75NTR (1 : 200, Abcam, Cambridge, UK), and anti-ROCKII (1 : 400, Abcam), respectively. The sections were incubated with goat anti-rabbit biotinylated IgG (1 : 200, Vector Laboratories, CA, USA) for 1 h at room temperature. After being incubated with HRP streptavidin (1 : 200, Vector Laboratories), immune complexes were visualized by incubation of sections in the DAB system. The sections were then coverslipped with neutral balsam as described above.

### 2.8. Detection of BDA-Labeled Fibers

Each segment from the BDA-labeled part was cut into serial, 25 *μ*m thick, parasagittal sections in the same cryostat for BDA tracing. BDA was visualized using the Neuro Trace™ BDA Neuronal Tracer Kit (Molecular Probes, Invitrogen, ON, Canada). Sections were rinsed several times in 0.01 M PBS and then incubated in the avidin-HRP working solution (0.2 *μ*g/ml) overnight at 4°C. The next day, the tissue sections were rinsed in PBS three times for 5 min each. DAB working solution (0.1% concentration with 0.03% H_2_O_2_) was applied to the sections under light microscope observation. After being terminated in distilled water, the sections then were air-dried, dehydrated, defatted, and coverslipped. An Olympus microscope (IX-81, Olympus, Japan) was used for photomicrography every third BDA-labeled sections intact in a bright field with the assistance of Volocity 6.0 software (PerkinElmer, USA) at 40x magnification. The BDA-labeled axons at the rostral and the caudal area within 1 mm from the edge of lesion epicenter (LEp) were counted and summed up as they were longer than 40 *μ*m [29]. The percentage of BDA-labeled axons passing through the LEp (*P*_BDA_) was calculated as *P*_BDA_ = (the sum of BDA fibers at caudal region/the sum of BDA fibers at rostral region) × 100%.

### 2.9. Stereological Analysis

A Leica microscope (DM6000B, Germany) with motorized stage and StereoInvestigator (Version 8.0 Software, MicroBrightField, USA) were used in stereological analysis. According to rules of the unbiased random sampling [30], starting sections were chosen at random and every eighth section throughout the entire injury segment was analyzed.

The volumes of lesion epicenter (*V*_LEp_) and spared tissue (*V*_SP_, including *V*_GM_ and *V*_WM_) were measured from MBP immunostained sections using the Cavalieri estimator probe in StereoInvestigator [31] and were calculated by the software [32]. MBP was used for presentation of myelin sheath [27, 33]. In the MBP stained tissue, the LEp was identified as an apparent cystic cavity, whose edge consisted of intensely stained nut-brown myelin deposits. Some damaged tissue in the cystic cavity lost normal neurons instead of clumps of small cells stained bright purple with darkly stained nuclei (reactive glia or gliosis). The spared grey matter (GM) was devoid of MBP but had normal neurons stained with cresyl violet. The spared white matter (WM) was MBP positive in grossly normal appearance, and there was a lack of dense gliosis (this could distinguish normal WT from the damaged tissue) [34]. We also analyzed the ratios (*R*) of *V*_LEp_, *V*_GM_, and *V*_WM_ to the volume of the total segment (*V*_*T*_), which were computed using the following expression to minimize the influence of tissue processing and staining methods: *R*_*X*_ = (*V*_*X*_/*V*_*T*_) × 100%.

The number of residual neurons (*N*_RN_) stained with cresyl violet in entire grey matter of spared tissue was quantified by the Optical Fractionator probe [35]. The sections used for quantitation were at the same segmental level in the three groups of rats injured. The size of the unbiased counting frame was 100 *μ*m × 100 *μ*m, and the space between the adjacent frames was 400 *μ*m. In order to distinguish neurons from glial cells in the quantitative analysis, only neurons with an ovoid, spherical, triangular, or multipolar profile and the soma diameter larger than 10 *μ*m with an intact cellular membrane and a clearly defined nucleus were counted [27]. The calculation of *N*_RN_ in the spare tissue was performed as previously described [32], and the average density of residual neurons (*D*_RN_, cells per mm^3^) was calculated in each group as *D*_RN_ = (*N*_RN_/*V*_SP_).

The positive cells of immunohistochemical staining for Nogo-A, p75NTR, and ROCKII were also demonstrated in stereological analyses as above, and the number of positive cells in the LEp and the total area was counted. To lessen the systematic error of tissue preparation procedure, we calculated the ratio of the number of positive cells in the LEp to that in the total area. To further analyze the distribution of the positive cells around the LEp area, we counted the number of positive cells in a range of 3 mm beyond the LEp outmost edge.

### 2.10. Statistical Analysis

All data were expressed as mean ± standard error (mean ± SEM) and analyzed using the software package SPSS 11.0 (SPSS, Inc., Chicago, IL, USA). Changes in BBB scores (*S*_BBB_) over time for 4 groups were analyzed using repeated-measures ANOVA with the between-groups factor, followed by Fisher's least significant difference (LSD)* post hoc* test. All stereological quantifications and *P*_BDA_ were analyzed using one-way ANOVA to compare differences among the four groups followed by Fisher LSD* post hoc* analysis. The significance was set at *P* < 0.05.

## 3. Results

### 3.1. Effects of CIG on Locomotor Function in SCI Rats

**Figure 2:**
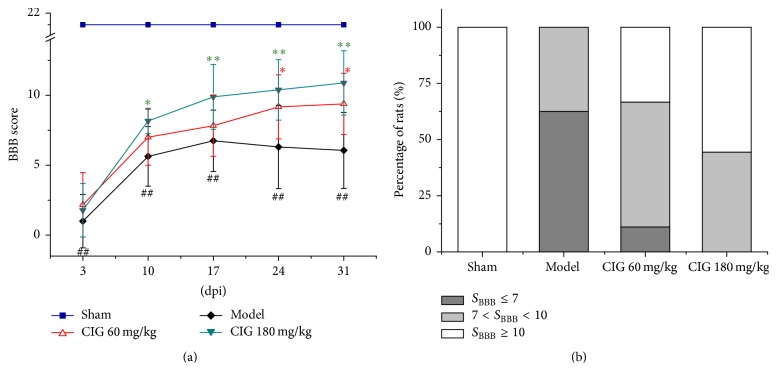
Effects of CIG on locomotor function in spinal cord injury (SCI) rats. Cornel iridoid glycoside (CIG) was intragastrically administered to rats starting at 4 h after the surgery of SCI and once a day for 31 days. The locomotor function was evaluated by Basso, Beattie, and Bresnahan (BBB) locomotor rating scale in the open-field test. (a) The BBB scores (*S*_BBB_) determined at 3, 10, 17, 24, and 31 days post-injury (dpi). Data represent mean ± SEM, *n* = 7–9. ^##^ *P* < 0.01, the SCI model group versus sham control group; ^*∗*^ *P* < 0.05, ^*∗∗*^ *P* < 0.01, CIG-treated groups versus the model group. (b) The percentages of rats with *S*_BBB_ ≤ 7, 7 < *S*_BBB_ < 10, and *S*_BBB_ ≥ 10 in total rats, respectively, at 31 dpi.

The rat locomotor function was evaluated by using the Basso, Beattie, and Bresnahan (BBB) locomotor rating scale in the open-field test.  Figure 2(a) shows the time course results of the BBB test at 3, 10, 17, 24, and 31 days post-injury (dpi) of the spinal cord in rats. BBB scores (*S*_BBB_) in the SCI model rats decreased significantly compared with those in sham-operated animals at all time points (*P* < 0.01). The intragastric administration of CIG at a dose of 60 mg/kg once a day starting at 4 h after the SCI surgery significantly elevated *S*_BBB_ at 24 and 31 dpi (*P* < 0.05). In the CIG 180 mg/kg-treated animals, *S*_BBB_ was significantly increased at 10, 17, 24, and 31 dpi (*P* < 0.05, *P* < 0.01) when compared with the SCI model rats.

According to the *S*_BBB_ interpretation [36], if *S*_BBB_ ≤ 7, the rat is likely to never walk with its hindlimbs again, which means the rat is completely uncured. By contrast, *S*_BBB_ ≥ 10 predicts that the rat has reached a level of recovery sufficient to walk. If *S*_BBB_ is between 7 and 10, the rat has a chance to eventually walk. Therefore, we calculated the percentages of rats in these three categories in all injured groups at 31 dpi.  Figure 2(b) displays that 62.5% of the rats had *S*_BBB_ ≤ 7 and no rat got *S*_BBB_ ≥ 10 in the SCI model group. However, in the CIG 60 mg/kg-treated group, 33.3% of the rats obtained *S*_BBB_ ≥ 10, 55.6% had *S*_BBB_ between 7 and 10, and only 11.1% presented *S*_BBB_ ≤ 7. In the CIG 180 mg/kg-treated group, 55.6% of the rats obtained *S*_BBB_ ≥ 10, and the others presented *S*_BBB_ between 7 and 10. The results demonstrated that CIG dose-dependently improved the locomotor impairment and promoted the functional recovery in SCI rats.

### 3.2. Effects of CIG on the Volume of Lesion Epicenter and Spared Tissue in the Injured Spinal Cord

**Figure 3:**
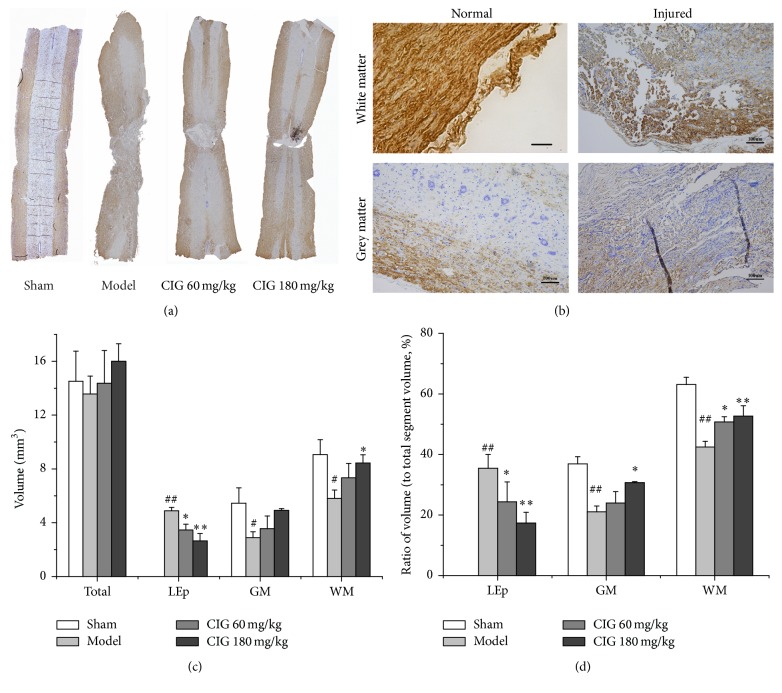
Effects of CIG on volume of lesion epicenter and spared tissue in injured spinal cord. Myelin basic protein (MBP) immunostaining with cresyl violet counterstaining was used to measure the stereological parameters of spinal cord lesion. (a) The representative coronary section of injured spinal cord in different groups. (b) The enlarged images of white and grey matter in normal and injured spinal cord. (c) The quantitative analyses for the volume of different parts of the spinal cord samples from actual stereological measurements. LEp, lesion epicenter; GM, grey matter; WM, white matter. (d) The ratio of the volume of different parts (*V*_LEp_, *V*_GM_, and *V*_WM_) to the total segment volume (*V*_*T*_). Data are expressed as mean ± SEM from 3 rats in each group. ^#^ *P* < 0.05, ^##^ *P* < 0.01, the SCI model group versus the sham control group; ^*∗*^ *P* < 0.05, ^*∗∗*^ *P* < 0.01, CIG-treated groups versus the model group.

In order to measure the SCI stereological parameters, myelin basic protein (MBP) immunostaining with cresyl violet counterstaining was used in this experiment. The representative coronary section of the injured spinal cord in the different groups is presented in Figure 3(a), while Figure 3(b) shows the enlarged images of white and grey matter in the normal and injured spinal cord. The statistical analysis of the actual stereological measurements for the volume of different parts of the spinal cord samples is presented in Figure 3(c). There was no significant difference among the groups in the volume of the total segment (*V*_*T*_), which meant that the tissue preparation procedure was consistent and the comparison of the other stereological parameters was on the same baseline. In the SCI model rats, the volume of the lesion epicenter (*V*_LEp_) significantly increased (*P* < 0.01), and the volume of the spared grey matter (*V*_GM_) and white matter (*V*_WM_) obviously decreased (*P* < 0.05). The treatment with 60 and 180 mg/kg CIG markedly reduced *V*_LEp_ (*P* < 0.05, *P* < 0.01) and increased the spared *V*_WM_ (*P* < 0.05) compared with the SCI model rats.

To minimize the systematic errors in the tissue preparation procedure, we calculated the ratio of the volume of different parts (*V*_LEp_, *V*_GM_, and *V*_WM_) to the volume of total segment (*V*_*T*_).  Figure 3(d) shows that the ratio of *V*_LEp_ to *V*_*T*_ (*R*_LEp_ = *V*_LEp_/*V*_*T*_) significantly increased (*P* < 0.01), and the ratios of *V*_GM_ and *V*_WM_ to *V*_*T*_ (*R*_GM_ = *V*_GM_/*V*_*T*_ and *R*_WM_ = *V*_WM_/*V*_*T*_) markedly decreased (*P* < 0.01) in the SCI model rats compared with those in the sham control animals. The treatment with 60 and 180 mg/kg CIG significantly reduced *R*_LEp_ (*P* < 0.05, *P* < 0.01) and elevated *R*_WM_ (*P* < 0.05, *P* < 0.01) in SCI rats; and the 180 mg/kg CIG treatment also obviously increased *R*_GM_ (*P* < 0.05). The results in Figure 3(d) are believed to be more accurate than those in Figure 3(c). These outcomes indicated that CIG decreased the damage of spinal cord in rats.

### 3.3. Effects of CIG on Residual Neurons in the Injured Spinal Cord

**Figure 4:**
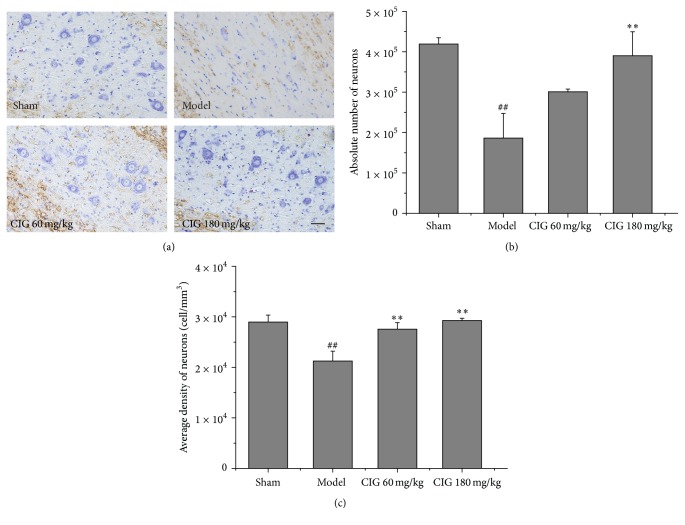
Effects of CIG on residual neurons in injured spinal cord. (a) The representative images of residual neurons stained by cresyl violet for Nissl substance. Scale bar = 50 *µ*m. (b) The quantitative analyses of stereological measurements for the absolute number of residual neurons (*N*_RN_) in the spared tissue (GM and WM) of injured spinal cord. (c) The average density of residual neurons (*D*_RN_, cells per mm^3^) in injured spinal cord. *D*_RN_ = *N*_RN_/*V*_SP_, in which *V*_SP_ represents the volume of spared tissue. Data are presented as mean ± SEM from 3 rats in each group. ^##^ *P* < 0.01, the SCI model group versus the sham control groups; ^*∗∗*^ *P* < 0.01, CIG-treated groups versus the model group.

The residual neurons measured by cresyl violet staining for Nissl substance are displayed in Figure 4(a). The statistical analysis of the stereological measurements for the absolute number of residual neurons (*N*_RN_) in the spared tissue (GM and WM) of the injured spinal cord is presented in Figure 4(b). *N*_RN_ significantly decreased in the SCI model rats compared with the sham-operated control rats (*P* < 0.01). CIG treatment dose-dependently increased *N*_RN_, in which there was a significant difference between the CIG 180 mg/kg-treated animals and the SCI model rats (*P* < 0.01).

For the purpose of minimizing the systematic error in the tissue preparation procedure, we calculated the average density of residual neurons (*D*_RN_ = *N*_RN_/*V*_SP_, in which *V*_SP_ represents the volume of spared tissue, including GM and WM).  Figure 4(c) exhibits that a significant decrease in *D*_RN_ was found in the SCI model rats (*P* < 0.01), and the 60 and 180 mg/kg CIG treatments obviously increased *D*_RN_ (*P* < 0.01) in SCI rats. The results in Figure 4(c) are believed to be more accurate than those in Figure 4(b). These outcomes demonstrated that CIG promoted neuron survival in the injured spinal cord.

### 3.4. Effects of CIG on BDA-Labeled Axons Passing through Lesion Epicenter in the Injured Spinal Cord

**Figure 5:**
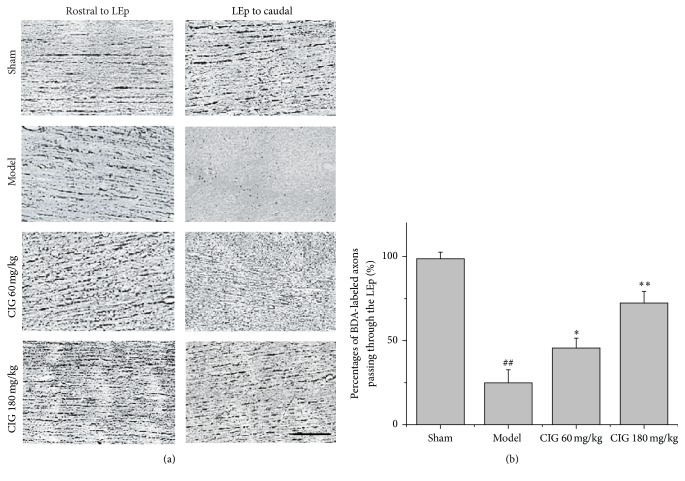
Effects of CIG on BDA-labeled axons passing through lesion epicenter in injured spinal cord. For the purpose of detecting the axon growth and passage through the lesion epicenter (LEp) in the spinal cord, BDA was injected into the sensorimotor cortex and the red nucleus at 10 dpi and then transmitted through the corticospinal tract and rubrospinal tract. (a) The representative images of BDA-labeled axons on parasagittal sections at the rostral and the caudal area within 1 mm from the edge of LEp in the injured spinal cord at 31 dpi are presented. Scale bar = 25 *μ*m. (b) The quantitative analysis for the percentage of BDA-labeled axons passing through the LEp. Data are presented as mean ± SEM from 3 rats in each group. ^##^ *P* < 0.01, the SCI model group versus the sham control groups; ^*∗*^ *P* < 0.05, ^*∗∗*^ *P* < 0.01, CIG-treated groups versus the model group.

In order to investigate the axon growth and passage through the LEp in the spinal cord, BDA was injected into the sensorimotor cortex and the red nucleus at 10 dpi and then transmitted through the corticospinal tract (CST) and rubrospinal tract (RST).  Figure 5(a) displays the representative images of BDA-labeled axons in parasagittal sections of the injured spinal cord at 31 dpi. In the rostral area 1 mm to the LEp, all groups had the intact BDA-labeled fibers, and their shapes were very much alike. In the caudal area 1 mm to the LEp, the BDA-labeled axonal fibers obviously decreased in the SCI model rats, indicating the interruption of axonal transport; but the treatment with CIG 60 and 180 mg/kg increased the BDA-labeled fibers after SCI.  Figure 5(b) shows that the percentage of BDA-labeled axons passing through the LEp (*P*_BDA_) significantly decreased in the SCI model group compared with that in the sham-operated rats (*P* < 0.01). The treatments with 60 and 180 mg/kg CIG obviously increased *P*_BDA_ in SCI rats (*P* < 0.05, *P* < 0.01). These results suggest that CIG may promote axon growth and passage through the LEp, implying the functional recovery of axonal transport after SCI.

### 3.5. Effects of CIG on Nogo-A, p75NTR, and ROCKII Expression in the Injured Spinal Cord

**Figure 6:**
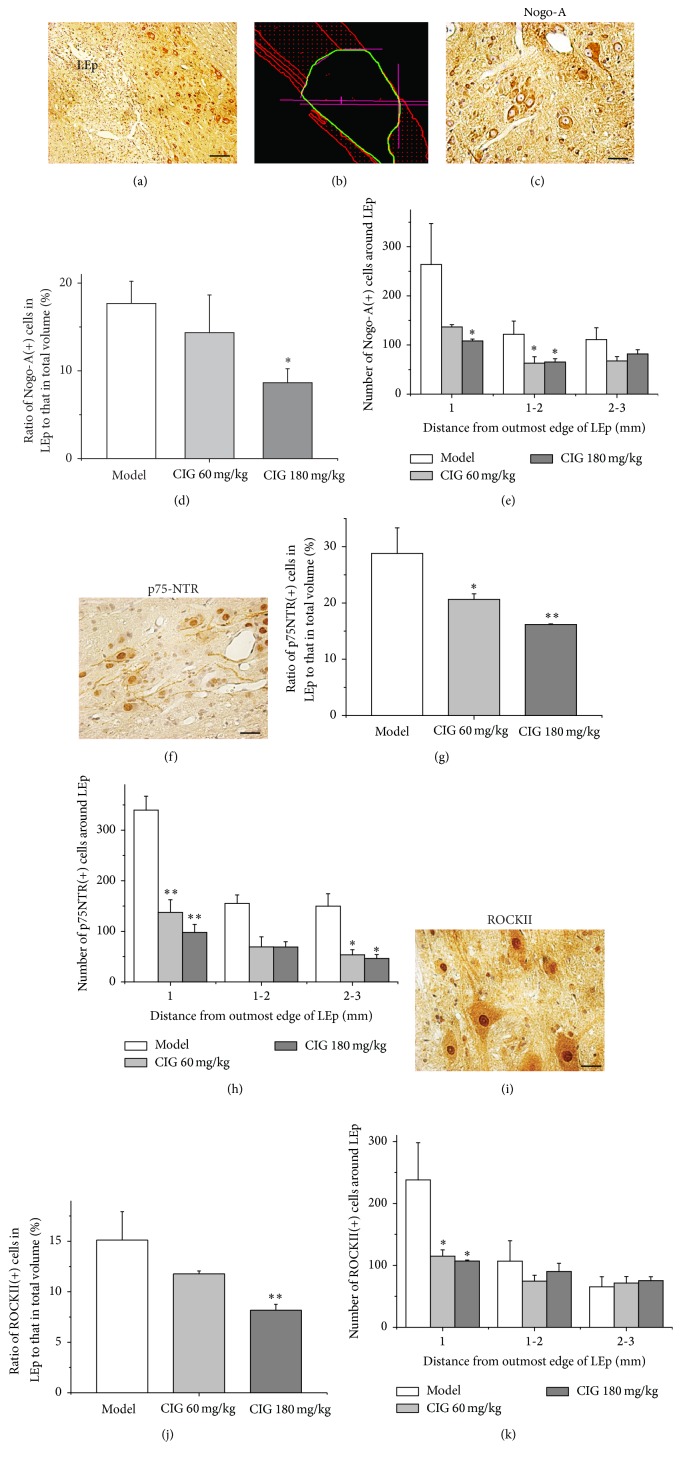
Effects of CIG on expression of Nogo-A, p75NTR, and ROCKII in injured spinal cord. The immunohistochemical and stereological methods were used to measure the expression of Nogo-A, the p75 neurotrophin receptor (p75NTR), and Rho-associated coiled-coil forming protein kinase II (ROCKII) on the serial and equal interval slices. (a) The lesion epicenter (LEp) and adjacent area stained by immunohistochemical method. Scale bar = 500 *μ*m. (b) The LEp and adjacent area analyzed by stereological method. The representative images of Nogo-A (c), p75NTR (f), and ROCKII (i) positive cells were shown, respectively. Scale bar = 50 *μ*m. (d, g, j) The ratio of the number of Nogo-A, p75NTR, and ROCKII positive cells in the LEp to that in the total volume, respectively. (e, h, k) The number of Nogo-A, p75NTR, and ROCKII positive cells around the LEp (within 3 mm from the outmost edge of LEp), respectively. Data are expressed as mean ± SEM from 3 rats in each group. ^*∗*^ *P* < 0.05, ^*∗∗*^ *P* < 0.01, CIG-treated groups versus the SCI model group. Because there was no LEp in the sham control rats, the boundary of LEp could not be distinguished; thus the sham control group was absent in this figure.

The expression of Nogo-A, p75NTR, and ROCKII in the myelin-associated inhibition signaling pathway was measured by immunohistochemical and stereological methods.  Figure 6 displays that CIG treatment (180 mg/kg) significantly decreased the ratios of Nogo-A, p75NTR, and ROCKII positive cells in the LEp to those in the total volume when compared with the SCI model rats, respectively (*P* < 0.05, *P* < 0.01); and CIG (60 mg/kg) also declined this ratio for p75NTR (*P* < 0.05). Around the LEp, CIG treatment (60 and 180 mg/kg) obviously reduced the number of Nogo-A, p75NTR, and ROCKII positive cells in a range of 1–3 mm beyond the outmost edge of LEp, respectively (*P* < 0.05, *P* < 0.01). These results demonstrated that CIG treatment inhibited the expression of Nogo-A, p75NTR, and ROCKII both in the LEp and around the LEp of the injured spinal cord, suggesting a downregulation effect on the myelin-associated inhibition signaling pathway.

## 4. Discussion

The present study indicated that CIG treatment improved the locomotor impairment, decreased the LEp volume, preserved more spared tissue and residual neurons, promoted more biofunctional axons passing through the LEp, and reduced Nogo-A, p75NTR, and ROCKII expression in the injured spinal cords of rats.

The BBB scale is considered to be an effective and predictive measure to recognize behavioral outcomes due to different injuries and to predict anatomical alterations at the lesion site [26]. It has therefore been widely and well accepted as a tool in therapeutic intervention development [37]. In the present study, we used the BBB scale and found that intragastric administration of CIG starting at 4 h after SCI surgery improved the locomotor impairment in rats. These results suggest that CIG may have the potential to ameliorate the clinical locomotor symptoms in patients with SCI.

The stereological approach provides an objective unbiased assessment of the structural changes in biological systems and it has been used to analyze neuroanatomical parameters of the spinal cord for a decade [38]. In the SCI model, the loss of axons and WM damage as well as the loss of neurons and GM damage contribute to the locomotor deficits [39, 40]. Earlier studies on SCI in rats have demonstrated that, with decreased size of the LEp and increased WM sparing, better behavioral function is observed in the open-field locomotor test [27, 41]. The correlation between WM sparing and locomotor function is also confirmed by other pharmacological studies [42, 43]. In the present study, CIG treatment reduced the LEp volume and preserved more spared tissue, including GM and WM. This might be one of the mechanisms by which CIG treatment improved the locomotor impairment in the SCI rats.

As a functional and anatomical unit, neurons of the spinal cord are the final common pathway for the transmission of the neuronal information from a variety of sources to the skeletal muscles [44]. Besides the immediate cell death after SCI, the secondary injuries, including ischemia, excitotoxicity, glial activation, and inflammatory processes, cause progressive degeneration and neuron death [5, 6]. In the present study, CIG treatment increased the number of residual neurons in the injured spinal cord. This is consistent with our previous studies on the brain damage. In those studies, CIG treatment increased the survival neurons in the brain, through inhibiting glial cell activation and inflammation and promoting neurotrophic factor expression and neurogenesis, in traumatic brain injury rats [45] and fimbria-fornix transaction-induced brain injury rats [19] as well as in focal cerebral ischemia rats [17, 18]. Since both the brain and the spinal cord belong to the central nerve system, the results of CIG from our studies on the brain damage might be helpful to explain the mechanisms by which CIG increased neuron survival in the injured spinal cord.

It is widely believed that functions are impaired or completely lost depending on the severity of the axonal tract damage, while function recovery relies on the restoration of the corresponding impaired axons [46]. The CST and RST are the two major long descending tracts in the dorsal and lateral funiculi of the spinal cord. Both of them are involved in skilled motor functions such as stride length [47]. Strategies have been elaborated to stimulate neurons to regenerate damaged axons when uninjured fibers can contribute to the formation of new functional circuitry by sprouting collaterals [48, 49]. It has been reported that the RST and CST regeneration can enhance the recovery of the locomotor function [50, 51]. In the present study, we labeled the CST and RST with BDA and found that CIG facilitated BDA-labeled fibers passing through the LEp in the spinal cord. This implies that CIG might promote axon growth or regeneration, which allows the transmission of biochemical molecules and signals in the caudal region of the spinal cord. This might be another mechanism by which CIG improved locomotor impairment in SCI rat.

To further explore the mechanism of CIG-induced axon growth, certain important molecules (such as Nogo-A, p75NTR, and ROCKII) in the myelin-associated inhibition signaling pathway were measured in this study. The axonal growth cone collapse induced by inhibitory molecules associated with myelin is one of the major causes for the inhibition of the axon regeneration [7, 52]. Nogo-A is a myelin-associated neurite outgrowth inhibitor [53]. Anti-Nogo antibodies have been shown to improve function recovery after SCI in rodents [54, 55]. p75NTR is upregulated after injury [56] and plays a role in signal transduction and neuronal apoptosis [10]. An inhibitor of the Nogo receptor and p75NTR complexes presented some benefits in a clinical trial [57]. As an important downstream effector, ROCKII is an attractive target for therapies [13]. Promising strategies targeting ROCKII by small molecule inhibitors and RNAi approaches are evaluated and some of them promote the recovery from the injury [12]. In the present study, we found that CIG treatment reduced Nogo-A, p75NTR, and ROCKII expression both in the LEp and around the LEp of the injured spinal cord, indicating that CIG downregulated the myelin-associated inhibition signaling pathway. This might be one of the mechanisms by which CIG promotes axon growth in the injured spinal cord.

In conclusion, the current study demonstrated that intragastric administration of CIG ameliorated locomotor impairment, reduced the LEp volume, increased the spared tissue (GM and WM) and neuron survival, enhanced biofunctional axons growth and their passage across the lesion site, and downregulated Nogo-A, p75NTR, and ROCKII expression in the myelin-associated inhibition signaling pathway in the injured spinal cords in rats. The results suggest that CIG may be beneficial to SCI treatment.
